# Antioxidant and Anti-Aging Properties of Polyphenol–Polysaccharide Complex Extract from *Hizikia fusiforme*

**DOI:** 10.3390/foods12203725

**Published:** 2023-10-10

**Authors:** Shangkun Li, Yunhai He, Saiyi Zhong, Yutong Li, Yuan Di, Qiukuan Wang, Dandan Ren, Shu Liu, Di Li, Fangjie Cao

**Affiliations:** 1 College of Food Science and Engineering, Dalian Ocean University, Dalian 116000, China; 18736192663@163.com (S.L.); 13848442033@163.com (Y.L.); 17866559577@163.com (Y.D.); wqk320@dlou.edu.cn (Q.W.); rdd@dlou.edu.cn (D.R.); liushu@dlou.edu.cn (S.L.); ldiss42@163.com (D.L.); cfj18226792740@163.com (F.C.); 2 Guangdong Provincial Key Laboratory of Aquatic Product Processing and Safety, Guangdong Ocean University, Zhanjiang 524088, China; 3 Key Laboratory of Aquatic Product Processing and Utilization of Liaoning Province, Dalian Ocean University, Dalian 116023, China; 4 National R&D Branch Center for Seaweed Processing, Dalian Ocean University, Dalian 116023, China

**Keywords:** *Hizikia fusiforme*, polyphenol–polysaccharide complex, antioxidation, anti-aging, gut microbiota

## Abstract

*Hizikia fusiforme* has a long history of consumption and medicinal use in China. It has been found that natural plants containing polyphenol–polysaccharide complexes have better activity compared with polyphenols and polysaccharides. Therefore, in this study on enzymatic hydrolysis and fractional alcohol precipitation, two kinds of polyphenol–polysaccharide complexes (PPC), PPC1 and PPC2, were initially obtained from *Hizikia fusiforme*, while the dephenolization of PPC1 and PPC2 produced PPC3 and PPC4. Through in vitro assays, PPC2 and PPC4 were found to have higher antioxidant activity, and thus were selected for testing the PPCs’ anti-aging activity in a subsequent in vivo experiment with D-gal-induced aging in mice. The results indicated that PPCs could regulate the expressions of antioxidant enzymes and products of oxidation, elevate the expressions of genes and proteins related to the Nrf2 pathway in the mouse brain, enrich the gut microbiota species and increase the *Bacteroidota*–*Firmicute* (B/F) ratio. Above all, the *Hizikia fusiforme* polyphenol–polysaccharide complex has potential in the development of natural anti-aging drugs.

## 1. Introduction

In recent years, improved living standards have increased people’s life expectancy [1]. Some studies have shown that by 2060, the elderly aged over 65 will account for 25% of the world’s population [2]. As people grow older, anti-aging has become a major concern of society and the scientific community. Aging is one of the risk factors that drive chronic diseases such as Alzheimer’s disease (AD), Parkinson’s disease (PD), Huntington’s disease (HD), cancers, etc. [3,4]. Relevant studies have revealed that oxidative stress is one of the leading causes of aging. Oxidative stress is a negative effect generated by free radicals in the body. It occurs from an imbalance between oxidants and antioxidants that is in favor of the oxidants, in which an excessive number of oxidative intermediates are produced. Compared with other organs, the brain is more susceptible to oxidative stress due to (1) its high content of peroxidizable unsaturated fatty acids, (2) its high oxygen consumption per unit of weight, (3) the high content of key components of lipid peroxidation and (4) the lack of an antioxidant defense system. Oxidative stress is considered to be the primary cause of several age-associated diseases [5].

Owing to their safety and no side effects, natural extracts have become a hot area in in anti-aging research. Cheng et al. found that polysaccharides extracted from Chinese *Radix sinensis* may delay aging in D-gal-induced Nestin-GFP mice, enhance antioxidant and anti-inflammatory ability and upregulate p53/P21 signaling [6]. Dong et al. stated that a flavonoid (secretin) could protect mice from D-gal-induced cognitive dysfunction and neuronal apoptosis by activating the NrF2-ARE signaling pathway [3].

Natural polyphenols and polysaccharides have many activities [7]. With the deepening of research, various approaches have been identified to prepare polyphenol–polysaccharide conjugates [8]. Polyphenol and polysaccharide complexes have better physicochemical properties and activities than when they are separate [9]. While past research into natural polyphenol–polysaccharide complexes has been insufficient, in recent years, people have begun to obtain polyphenol–polysaccharide complexes from plant extracts [10], which are believed to be safer than synthetic complexes. Studies on natural polyphenol–polysaccharide complexes have focused on terrestrial plants, and have found that these complexes have anticoagulant [11], antioxidant [12], antiradical [13], antitussive, and bronchodilation [14] activities; however, studies on their other activities have been insufficient.

*Hizikia fusiforme*, which is known as a macrophyte, is a species of perennial brown algae growing on the coastlines of China, Japan, South Korea, and other Asian countries. It belongs to the Chlorophyta of the Sargasso family [15]. China is a big producer of this plant, with more than 30,000 tons raised, as it has been regarded as a traditional Chinese medicine and food since ancient times [16]. It is an excellent source of polysaccharides, proteins, minerals and fats [17]. Studies have shown that polysaccharide and polyphenol extracts of *Hizikia fusiforme* have strong antioxidation and antitumor activities, and can improve the composition of intestinal flora [18,19].

In this study, polyphenol–polysaccharide complexes were prepared via enzymolysis with *Hizikia fusiforme* as the raw material, and another two dephenolized PPCs were prepared by oxidation with hydrogen peroxide. Their chemical composition and structural characteristics were analyzed individually, and their antioxidant and anti-aging activities were explored through in vitro and animal experiments. This study further extends the existing research on the activity of polyphenol–polysaccharide complexes and natural anti-aging substances.

## 2. Materials and Methods

### 2.1. Materials

*Hizikia fusiforme* was purchased from the National Seaweed Research and Development Center, Zhejiang, China. *Hizikia fusiform* was identified by Professor Zhang Zeyu, a renowned expert on algae from Dalian Ocean University, and the voucher specimens (DLOU-22.06.017) preserved in the functional room of Seaweed Resources Development and Utilization of Dalian Ocean University. Monosaccharide standards (fucose, galactose, mannose, glucuronic acid, galacturonic acid, xylose, rhamnose and glucose), DPPH, TPTZ, Tris, phthalide and D-gal were purchased from Sigma-Aldrich Co., (St. Louis, MO, USA). Other reagents not mentioned here were of analytical grade.

### 2.2. Extraction of Polyphenol–Polysaccharide Complex (PPC) from Hizikia fusiforme

Polyphenol–polysaccharide complexes were prepared using enzyme-assisted extraction. Briefly, 10 g of *Hizikia fusiforme* powder was soaked in 50 mL of deionized water for 20 min prior to centrifugation. The solid was collected and, together with 20 mg of cellulase, was immersed in 50 mL of deionized water. After 50 min in a water bath with continuous magnetic stirring, 5 g of sodium bicarbonate was added, and the mixture was again placed in the water bath at 50 °C for 50 min. Supernatant 1 was obtained by centrifugation. The precipitate was collected and put into 50 mL of deionized water with 3 g of sodium bicarbonate, then subjected to 50 min of magnetic stirring at 50 °C, followed by centrifugation to obtain Supernatant 2. Supernatants 1 and 2 were mixed, and the pH was adjusted to neutral to obtain the solution of the raw materials.

Next, 95% ethanol was added to the liquid until the concentration of ethanol reached 30% (*v*/*v*). After 20 min of resting, the suspension was placed in a centrifuge to collect the precipitate. The resulting solid was freeze-dried to obtain polyphenol–polysaccharide Complex 1 (PPC1). In addition, 95% ethanol was mixed into the supernatant until the concentration of ethanol in the supernatant was 70% (*v*/*v*). Similarly, after 20 min of resting, the suspension was centrifuged to collect the precipitate, which was also freeze-dried to obtain polyphenol–polysaccharide Complex 2 (PPC2).

### 2.3. Preparation of the Dephenolization Products of the Polyphenol–Polysaccharide Complex (PPC) of Hizikia fusiforme

According to the method of Usoltseva et al. [20], 100 mL of 10 mg/mL PPC1 and PPC2 were added to 20 mL of 30% H_2_O_2_, and the pH was adjusted to 8.5 with 10% ammonia. The mixture was left to stand for 24 h away from the light, centrifuged to collect the supernatant, dialyzed for 48 h (with a molecular weight cutoff of 3.0 kDa) and freeze-dried. The dialyzed form of PPC1 was named polyphenol–polysaccharide Complex 3 (PPC3), and the dialyzed form of PPC2 was named polyphenol–polysaccharide Complex 4 (PPC4).

### 2.4. Chemical Composition and Structure

#### 2.4.1. Chemical Analysis

In this study, the phenol–sulfuric acid method was used to determine the total sugar content of each component, with L-fucose:D-galactose = 3:1 as the standard [21]. The linear range spanned from 0 mg/mL to 250 mg/mL, and the correlation coefficient was R^2^ = 0.9976. To determine the total phenol content of the sample, the folinol method was used, with phloroglucinol as the standard [22]. The linear range spanned from 0 μg to 2.5 μg, and the correlation coefficient was R^2^ = 0.9994. The content of sulfate radical was determined via the barium chlorine–gelatin method, using K_2_SO_4_ as the standard [23]. The linear range spanned from 0 μg to 120 μg, and the correlation coefficient was R^2^ = 0.9981.

#### 2.4.2. Analysis of the Monosaccharide Composition

The analysis of the monosaccharide composition was conducted with Shimadzu equipment (GC-2010, Japan), following the method of Sheng et al. [24] with a slight modification. The standard monosaccharides included fucose (Fuc), mannose (Man), xylose (Xyl), rhamnose (Rha), glucose (Glu), galactose (Gal), galactose acid (Glu-UA) and glucuronic acid (Gal-UA). The monosaccharides’ molar ratios were calculated by comparing the standard monosaccharides, and with area normalization.

#### 2.4.3. FT-IR Spectroscopy

In line with the method of Jafari et al. [25], 1 mg of the PPCs was mixed with 100 mg of KBr, ground and pressed, and analyzed using a Fourier transform infrared spectrometer (Invenio, Bruker, Saarbrucken, Germany), with a scanning wavelength of 4000–400 cm^−1^.

### 2.5. Determination of Antioxidant Activity

The DPPH-scavenging activity was determined via an earlier method with minor modifications [26]. The activity of scavenging hydroxyl radicals was measured via the method of Cichewicz et al., with a few modifications [27]. The activity of superoxide anion scavenging was tested via the method of Tian et al., subject to moderate adjustments [28]. The ferric-reducing ability of plasma (FRAP) assay was carried out according to the practice of Tierney et al., also with some refinements [26].

### 2.6. Determination of the Mixture Effects

According to the method of Obluchinskaya et al. [29], with appropriate modifications, the mixture effects (ME) of PPC1 and PPC2 were calculated. The antioxidant power (AOP) was calculated as AOP = 1/IC_50_ (FRAP: 1/concentration required to reach 0.4 mmol of reducing power). The ME was defined by comparing the experimental AOP (*AOP_exp_*) of PCC1 and PPC2 with the AOP (*AOP_cale_*) calculated from the sum of PPC3 and PPC4, and the efficiencies of phloroglucinol relative to their proportions in the mixture. The equation was as follows:

$$ME=\frac{{AOP}_{exp}}{{AOP}_{cale}}$$

A value *ME* > 1 indicates synergy, whereas a value *ME* < 1 indicates antagonism and a value *ME* = 1 means neither synergy nor antagonism; each dataset was the average of three experiments.

### 2.7. Animals

#### 2.7.1. Animal Groupings

Clean Kunming mice weighing 20 ± 2 g were purchased from Liaoning Changsheng Biotechnology Co., Ltd. (Shenyang, China). The mice were randomly divided into 9 groups, with 12 in each group, and administered the treatments for 37 consecutive days. The mice in the normal group (NC) were fed with distilled water by gavage and normal saline by intraperitoneal injection. The mice in the model group (MC) were fed with water by gavage and given 100 mg/kg D-gal by intraperitoneal injection. Mice in the positive group (PC) were given vitamin E (VE) (50 mg/kg) by gavage and D-gal (100 mg/kg) by intraperitoneal injection. The mice in the PPC2-L group were administered with PPC2 (100 mg/kg) intragastrically and D-gal (100 mg/kg) intraperitoneally. The mice in the PPC2-M group were given PPC2 (300 mg/kg) intragastrically and D-gal (100 mg/kg) intraperitoneally. The mice in the PPC2-H group were fed with PPC2 (500 mg/kg) intragastrically and given D-gal (100 mg/kg) intraperitoneally. The mice in the PPC4-L group were given PPC4 (100 mg/kg) intragastrically and D-gal (100 mg/kg) intraperitoneally. The mice in the PPC4-M group were administrated PPC4 (300 mg/kg) intragastrically and D-gal (100 mg/kg) intraperitoneally. The mice in the PPC4-H group were given PPC4 (500 mg/kg) intragastrically and D-gal (100 mg/kg) intraperitoneally. All of the mice were fasted for 12 h after the last administration, and then killed. The study was approved by the Ethical Committee for Experimental Animal Care at Dalian Ocean University (protocol code: DLOU20230610, 8 June 2023).

#### 2.7.2. Detection of Biochemical Indices in Serum and Liver Tissues

After the eyelid blood was collected and centrifuged at 3000 r/min for 15 min, the supernatant was measured to obtain the serum indices. The activity of superoxide dismutase (SOD), glutathione (GSH) (R^2^ = 0.9981; the linear range spanned from 0 μmol/mL to 100 μmol/mL), total antioxidant power (T-AOC), and malondialdehyde (MDA) in the mouse serum was determined in strict accordance with the instructions of the kit. A 10% liver homogenate was prepared and centrifuged at 3000 rpm and 4 °C for 15 min to obtain the supernatant. The activity of superoxide dismutase (SOD), glutathione peroxide (GSH-PX) (R^2^ = 0.9987; the linear range spanned from 0 μmol/mL to 100 μmol/mL), catalase (CAT), lipid peroxide (LPO) (R^2^ = 0.9997; the linear range spanned from 0 μmol/mL to 10 μmol/mL), and malondialdehyde (MDA) in the mouse livers were determined according to the instructions of the kit. All of the reagent kits were purchased from Nanjing Jianchen Bioengineering Institute (Nanjing, China). The multifunctional enzyme labeling apparatus utilized in the experiment was from Danli Technology (SpectraMax^®^ ABS Plus, Foshan, China).

#### 2.7.3. Quantitative PCR Fluorescence Analysis

The total mRNA of mouse brain tissue was extracted with the Trizol kit (Thermo Fisher, Shanghai, China), and the mRNA was transcribed into cDNA with a commercial kit (Aikorei Biology, Wuhan, China). The primers were designed as shown in Table 1, and the mRNA expression levels were quantified via RT-PCR (Roche, Shanghai, China), with the glycer-aldehyde-3-phosphate dehydrogenase (GAPDH) gene as an internal reference. The reaction conditions of the RT-PCR were as follows: 1 cycle at 95 °C for 30 s, 40 cycles at 95 °C for 5 s, 50 cycles at 60 °C for 1 min and 80 cycles at 95 °C for 15 s.

#### 2.7.4. Western Blot Analysis

The total protein (approximately 0.1 g) was extracted from brain tissues using a protein extraction kit according to the manufacturer’s instructions. The concentration of protein was measured using BCA protein assay kits. The proteins were separated with 4–20% SDS-PAGE and transferred onto PVDF membranes (Millipore, Burlington, MA, USA). Each membrane was blocked with 5% skim milk for 1 h, and then incubated overnight at 4 °C with the primary antibodies for Nrf2, HO-1, NOS, Nqo1, SOD1 and SOD2. After washing three times, the membrane was incubated with HRP-conjugated secondary antibodies for 30 min at room temperature. The protein bands were visualized using the ECL system and quantified by AlphaEaseFC™ software (Version 4.0.0, Alpha Innotech Corp., San Leandro, CA, USA). β-actin was used as the internal control for normalizing the expressions of the proteins.

#### 2.7.5. Sequencing and Analysis of the Gut Microbiota

The total DNA was extracted from the mouse fecal samples with the PowerMag™ Microbiome RNA/DNA Isolation (MOBIO, San Mateo, CA, USA) kit, and the quality was evaluated using 1% agarose gel electrophoresis. The V3–V4 region of 16Sr DNA was amplified. The resulting PCR products were purified and quantified for sequencing on the Illumina MiSeq platform.

The raw reads were filtered with Trimmomatic and merged by FLASH. The operational taxonomic units (OTUs) were clustered using the de novo OTUs selection protocol with a cutoff of 97% similarity. The taxonomy of the 16S rDNA gene sequences was determined by the RDP classifier algorithm, using the Silva database with a confidence threshold of 70%. Alpha diversity analysis, including the species observed and the Shannon, Chao1, and Simpson indices, was performed.

### 2.8. Statistical Analysis

In the animal experiments, two independent variables were used to design the experiments. The first variable was an intraperitoneal injection of saline versus an intraperitoneal injection of D-galactose, and the second variable was a gavage of saline, VE, a polyphenol–polysaccharide complex, or a dephenolization polyphenol–polysaccharide complex. Nine different groups of animal experiments were designed, and the dependent variables were the indices of the mice.

All data were expressed as the mean ± SD. One-way analysis of variance (ANOVA) and Duncan’s multiple comparison test were used for the statistical analyses, with *p* < 0.05 indicating a statistically significant difference (SPSS Statistics 21.0, IBM, New York, NY, USA).

## 3. Results

### 3.1. Chemical Composition of PPCs

**Figure 1:**
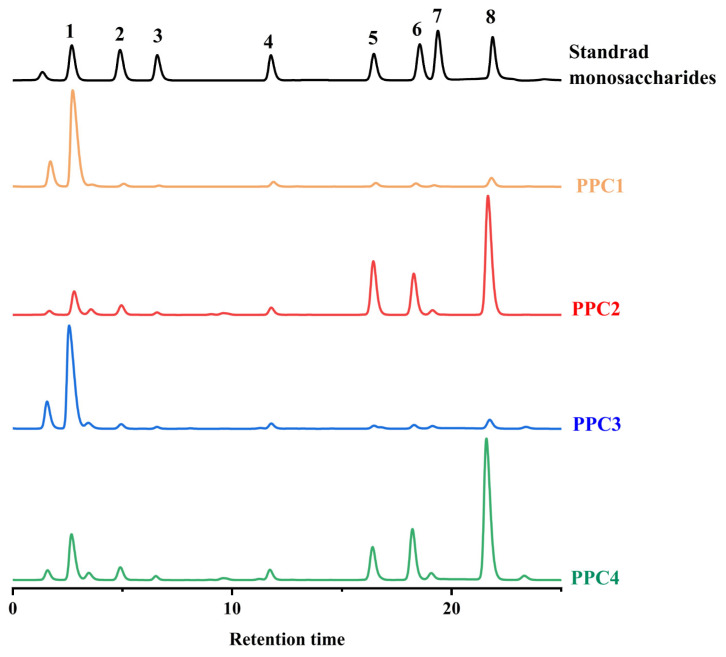
GC chromatograms of PPC1, PPC2, PPC3, PPC4 and the standard monosaccharides. The standard monosaccharides included (1) mannose, (2) rhamnose, (3) glucuronic acid, (4) galactose acid, (5) glucose, (6) galactose, (7) xylose and (8) fucose.

It can be seen from Table 2 that the two polyphenol–polysaccharide complexes (PPC1 and PPC2) were mainly composed of polyphenols, polysaccharides and sulfate groups, which represented more than 50% of the whole. The sugar content, total phenol content and sulfate content of PPC2 were higher than those of PPC1. According to the analysis of the monosaccharide composition shown in Figure 1, PPC1 was mainly composed of mannose (76.16%), while 64.52% of PPC2 was made up of fucose, glucose and galactose. The molar percentages of monosaccharides in PPC1 were not significantly different.

PPC3 and PPC4 were the oxidation products of PPC1 and PPC2. As can be seen from Table 2, the polyphenol content of PPC3 and PPC4 decreased to 0.77% and 1.62% after the oxidation treatment, respectively, while their sugar and sulfate content did not change significantly. The composition of the monosaccharides of PPC3 and PPC4 had no significant differences from the undephenolized PPCs. In addition, PPC4 had more fucose, mannose and galacturonic acid, and less glucose and glucuronic acid compared with PPC2, which may have been caused by the H_2_O_2_ treatment [20].

### 3.2. PPC FT-IR Spectroscopy Analysis

**Figure 2:**
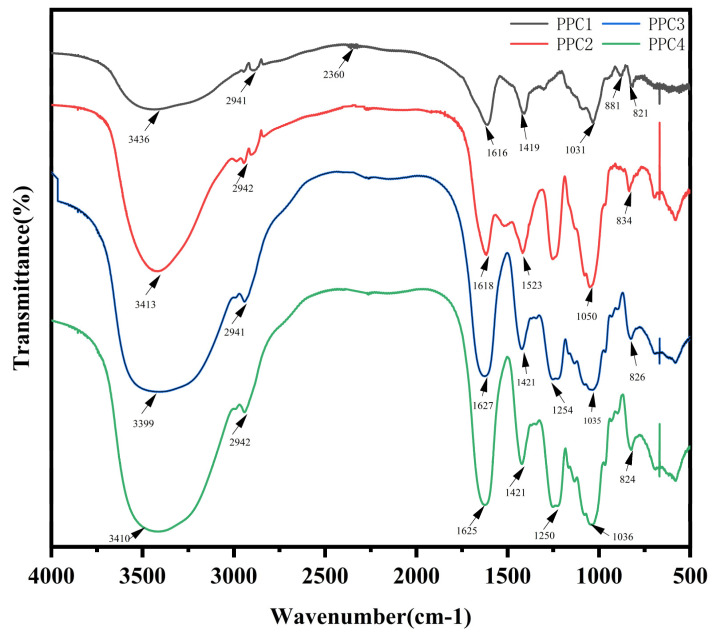
FT-IR spectra of PPC1, PPC2, PPC3 and PPC4.

The FT-IR results are shown in Figure 2. The absorption peaks of PPC1 and PPC2 appeared at about 3400 cm^−1^, which were O-H stretching vibration peaks [15]. Different degrees of absorption occurred at 3000–2800 cm^−1^ in all of the samples, particularly near 2990 cm^−1^ and 2940 cm^−1^. The peaks near 2990 cm^−1^ and 2940 cm^−1^ were caused by the C-H antisymmetric stretching vibration of the sugar ring and the methyl around polyphenol [294432]. The peaks of PPC1 and PPC2 at 2940 cm^−1^ were the weak methyl absorption peaks of fucose. There were strong absorption peaks near 1610 cm^−1^ for both PPC1 and PPC2, which were C=O double-bond stretching vibrations. The observed stretching vibration peak of C-O near 1419 cm^−1^ was a strong absorption peak, indicating the presence of uronic acid [30]. In addition, the peaks at 1270–1260 cm^−1^ signaled the presence of the stretching vibration of the sugar ring and the phenol structure (C=O=C) [31]. Both PPC1 and PPC2 showed obvious absorption peaks at 1050–1031 cm^−1^, which occurred because of the absorption of C-O [32]. The absorption peak near 820 cm^−1^ was the stretching vibration of the C-O-S equatorial coordination, indicating the presence of a small number of sulfate groups in the polyphenol–polysaccharide complexes [18]. PPC1 and PPC2 had absorption peaks at about 820 cm^−1^. These were due to sulphate radicals linked to the C2 or C3 position of fucose and the horizontal bond. The infrared spectra showed that the sulphate radicals of fucose were connected to C2 or C3 [33].

PPC3 and PPC4 had absorption peaks near 3400 cm^−1^. They also showed absorption peaks of different degrees at 3000–2800 cm^−1^, especially near 2990 cm^−1^ and 2940 cm^−1^, including a weak methyl absorption peak of fucose. Both PPC3 and PPC4 showed strong absorption peaks near 1620 cm^−1^ and C=O double-bond stretching vibrations. The peak of the C-O stretching vibration appeared near 1421 cm^−1^, which was a strong absorption peak, indicating the presence of uronic acid. Absorption peaks of PPC3 and PPC4 appeared near 1250 cm^−1^, which were signals of the stretching vibration of the sugar ring and the phenol structure (C=O=C). Both PPC3 and PPC4 showed obvious absorption peaks near 1035 cm^−1^, which were the stretching vibration peaks of C-O. The absorption peak near 820 cm^−1^ was the stretching vibration of C-O-S equatorial coordination, indicating that the polyphenol–polysaccharide complexes had a small amount of sulfate groups, which was consistent with the results regarding the content of the sulfate group. PPC3 and PPC4 both had absorption peaks near 820 cm^−1^, and these were related to sulphate radicals connected to the C2 or C3 position of fucose and the horizontal bond. Within the infrared spectrum, the sulphate radical of this sugar was shown to be connected to C2 or C3 [34].

### 3.3. Antioxidant Activity In Vitro

**Figure 3:**
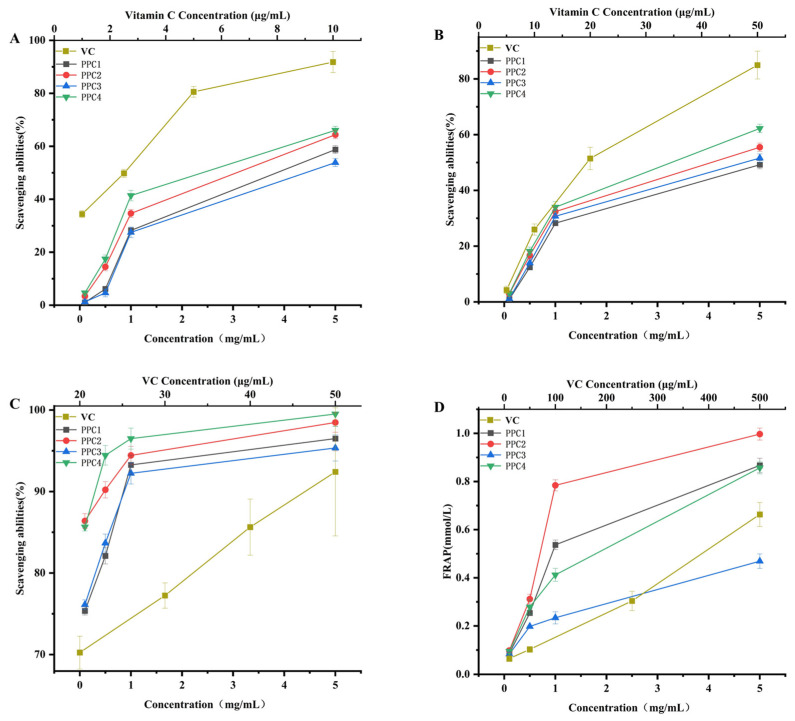
The in vitro antioxidant capacities of vitamin C, PPC1, PPC2, PPC3 and PPC4: DPPH free radical scavenging ability (**A**), hydroxyl radical scavenging ability (**B**), superoxide anion radical scavenging ability (**C**) and ferric ion reducing power (**D**).

As shown in Figure 3, the antioxidant activities of the four groups of samples were dose-dependent. When the concentration of the sample was 5 mg/mL, the capacity for scavenging free radicals of DPPH was as follows: PPC4 (66.24%) > PPC2 (63.58%) > PPC1 (57.66%) > PPC3 (53.54%). The hydroxyl radical scavenging ability was in the order PPC4 (62.24%) > PPC2 (55.49%) > PPC3 (51.61%) > PPC1 (49.23%). The results for the capacity of scavenging superoxide anions was PPC4 (99.52%) > PPC2 (98.48%) > PPC1 (96.5%) > PPC3 (95.35%). The reducing power, in decreasing order, was PPC2 (0.99 mmol/mL) > PPC1 (0.86 mmol/mL) > PPC4 (0.85 mmol/mL) > PPC3 (0.49 mmol/L). The vitamin C group showed similar antioxidant activity in the polyphenol–polysaccharide complexes with a lower concentration [33]. In this comprehensive analysis, the antioxidant capacity of PPC2 was always stronger than that of PPC1. For the dephenolized products, PPC4 performed better than PPC3 in terms of antioxidant activity, which was consistent with the untreated PPC1 and PPC2. PPC3 was inferior to PPC1, while PPC4 outperformed PPC2.

**Figure 4:**
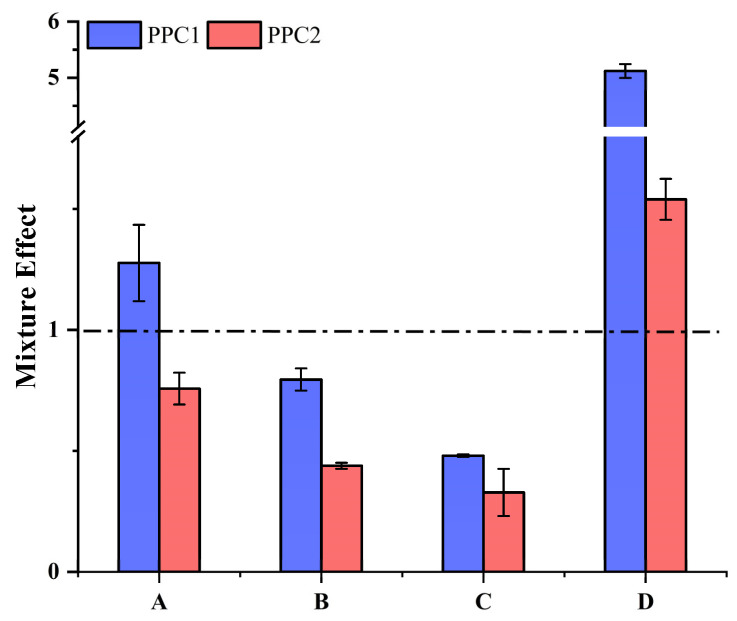
The mixture effect (ME) of the polyphenol polysaccharide complex (PPC1, PPC2): (**A**) DPPH free radical scavenging ability, (**B**) hydroxyl radical scavenging ability, (**C**) superoxide anion radical scavenging ability and (**D**) ferric ion reducing power.

In this study, the antioxidant activities of phloroglucinol were investigated, and the ME values were calculated by comparing phloroglucinol, the dephenolized polyphenol–polysaccharide complex and the original polyphenol–polysaccharide complex. As can be seen in Figure 4A, the ability to scavenge DPPH free radicals (PPC1: ME = 1.27; PPC2: ME = 0.75) indicated that phloroglucinol acted synergistically in PPC1 and antagonistically in PPC2. Both PPC1 and PPC2 had ME values of <1 for the hydroxyl radical as and superoxide anion radical scavenging activity, and both had ferric ion-reducing power ME values of >1. This proves that phloroglucinol plays different roles in the antioxidant activity of polyphenol–polysaccharide complexes.

### 3.4. Anti-Aging Activity of PPCs of Hizikia Fusiforme In Vivo

#### 3.4.1. Effects of PPC on the Serum and Liver Oxidation Indices of D-Gal-Induced Mice

**Figure 5:**
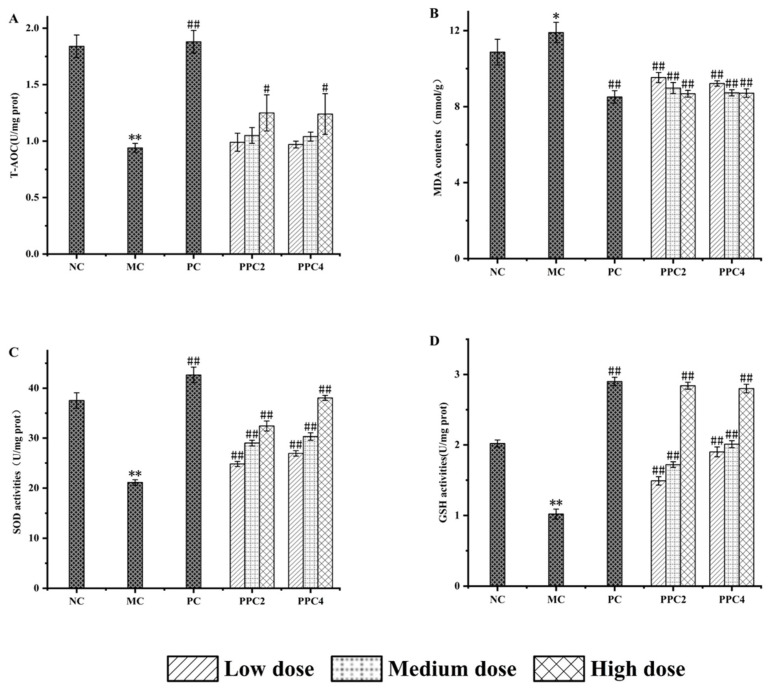
Effects of PPC2 and PPC4 on the T-AOC (**A**), MDA (**B**), SOD (**C**) and GSH (**D**) contents in sera of aging induced in mice with D-gal. The results are presented as the mean ± SD (n = 12). *: *p* < 0.05, **: *p* < 0.01, vs. NC group; #: *p* < 0.05, ##: *p* < 0.01, vs. MC group.

The values of various indices in sera from the mice are shown in Figure 5. Compared with the control group, the activities of T-AOC, GSH and SOD in the model group was significantly lower (*p* > 0.01), while the MDA content did not change significantly, indicating that the aging model had been successfully established [35]. In the different dosage groups, higher concentrations of PPC2 or PPC4 resulted in better levels of T-AOC, GSH and SOD, and less MDA. However, the PPC2-H and PPC4-H groups had different indices. Their performance in terms of T-AOC and MDA content was similar (*p* < 0.05), but significantly increased levels (*p* < 0.01) of SOD and GSH were detected in the PPC4-H group.

**Figure 6:**
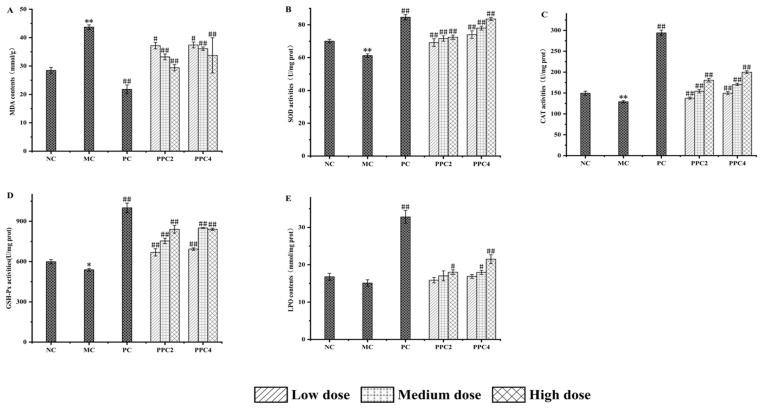
Effects of PPC2 and PPC4 on MDA (**A**), SOD (**B**), CAT (**C**), GSH-PX (**D**) and LPO (**E**) contents in the livers of aging mice induced by D-gal. The results are presented as the mean ± SD (n = 10). *: *p* < 0.05, **: *p* < 0.01, vs. NC group; #: *p* < 0.05, ##: *p* < 0.01, vs. MC group.

As shown in Figure 6, the MDA and GSH-PX contents in the model group increased compared with the control group (*p* < 0.01), while the activities of SOD, CAT and LPO decreased, indicating that the aging model had successfully been established [36]. The PPC2 and PPC4 groups had a dose-dependent effect on the liver indices. Higher doses yielded elevated activities of SOD, CAT and LPO, and a reduced MDA content. However, GSH-PX behaved differently in the two groups as the concentration of PPC2 and PPC4 increased. The effects of PPC2-H and PPC4-H on the different indices varied. There was no significant difference in the level of GSH-PX (*p* < 0.05), but, compared with PPC2-H, PPC4-H had significantly more SOD and CAT, less MDA and an improved LPO clearance rate.

#### 3.4.2. Effects of PPC on Brain-Related Genes in D-Gal-Induced Mice

**Figure 7:**
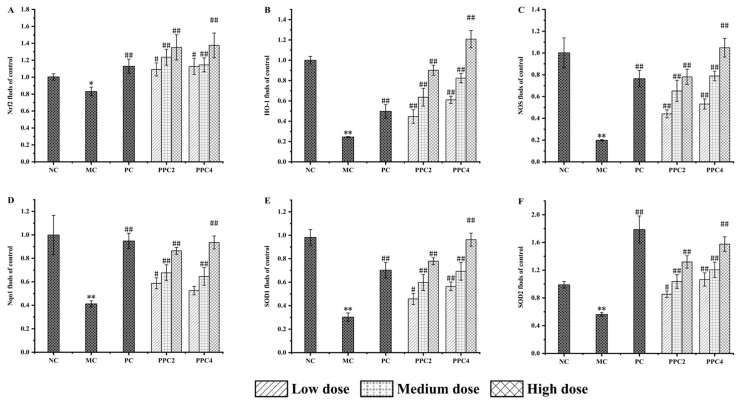
Effect of PPC2 and PPC4 treatment on cerebral mRNA. The results are presented as the mean ± SD (n = 12). Nrf2 (**A**); HO-1 (**B**); NOS (**C**); Nqo1 (**D**); SOD1 (**E**); SOD2 (**F**). *: *p* < 0.05, **: *p* < 0.01, vs. NC group; #: *p* < 0.05, ##: *p* < 0.01, vs. MC group.

The expressions of Nrf2, HO-1, NOS, Nqo1, SOD1 and SOD2 in the brains of the mice were investigated. As shown in Figure 7, compared with the blank group, the level of Nrf2 protein in the model group went significantly down, consistent with the changes in Nrf2 during the natural aging process of the animals [37]. Treatment with PPC2 and PPC4 enhanced the expression of Nrf2. This was more obvious in the high-dose group, in which the level of Nrf2 improved significantly in a dose-dependent manner. The changes in HO-1, Nqo1, NOS, SOD1 and SOD2 were basically consistent with those of Nrf2, which further proved the anti-aging effects of PPC2 and PPC4.

Compared with PPC4, PPC2 showed no significant differences in the levels of the Nrf2 and Nqo1 proteins in the NRF2-ARE gene pathway, but the levels of HO-1, NOS, SOD1 and SOD2 proteins were significantly different.

#### 3.4.3. Expressions of Nrf2, NOS, Nqo1, HO-1, SOD1 and SOD2 Proteins in the Brains of Mice

**Figure 8:**
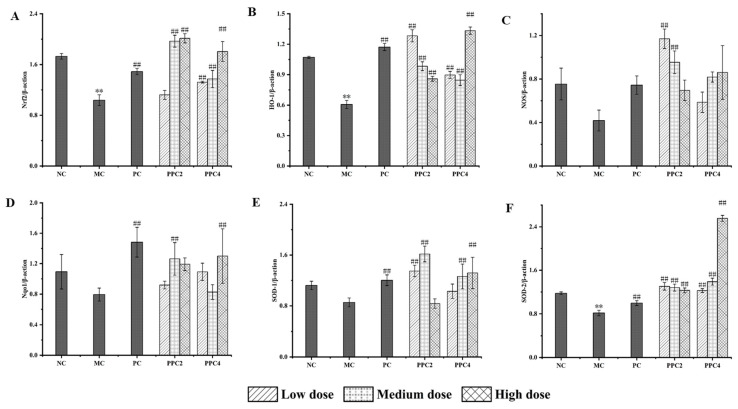
Effects of PPC2 and PPC4 on the expressions of cerebral proteins in aging mice. The results are presented as the mean ± SD (n = 12). Nrf2 (**A**); HO-1 (**B**); NOS (**C**); Nqo1 (**D**); SOD1 (**E**); SOD2 (**F**). **: *p* < 0.01, vs. NC group; ##: *p* < 0.01, vs. MC group.

An analysis of Figure 8 showed that in the MC group, the expressions of proteins related to the Nrf2 pathway in the brain of the mice decreased after the D-gal treatment [38], among which, NOS and HO-1 recorded the most significant losses, and the expression of the proteins improved after the VE treatment. The effects of PPC2 and PPC4 on protein expression levels were not consistent after oral gavage. More Nrf2 pathway proteins were expressed with an increase in the dose of PPC4, and the effect in the high-dose group was the most significant. In the PPC2 group, the expression levels of HO-1, NOS and SOD-2 proteins were the most significant in the medium- and low-dose groups. The expression levels of Nqo1 and SOD1 were the most significant in the medium-dose groups, and the expression of Nrf2 was significant in the high-dose group.

### 3.5. Sequencing and Analysis of the Gut Microbiota

**Figure 9:**
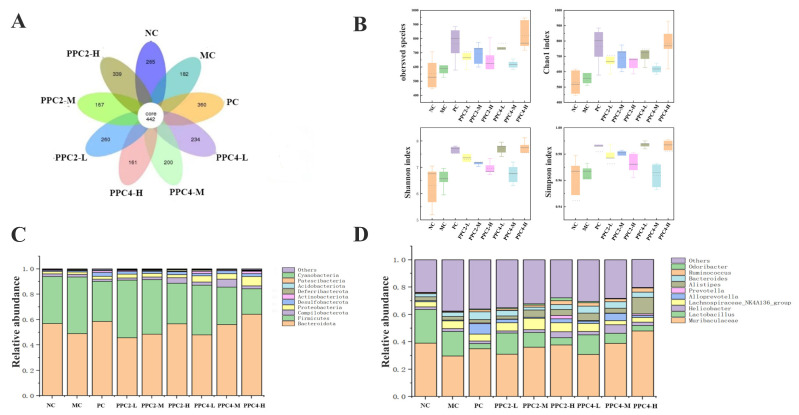
Effects of PPC2 and PPC4 on the abundance of species in the gut microbiota of D-gal-induced aging mice. Venn diagram of different groups, based on OTUs (**A**). Alpha diversity, including species observed, and the Shannon, Chao1 and Simpson indices (**B**). Community composition of the gut microbiota at the phylum level (**C**). Community composition of the gut microbiota at the genus level (**D**).

As shown in Figure 9A, 422 OTUs were observed in the nine groups, 265 of which were in the NC group. After D-gal induction, the number of OTUs in the MC group declined to 182, and that in the PPC4-H group was 339, which was the highest, except for the positive control group. In the PPC2-L and PPC2-M groups, the numbers of OTUs were 234 and 200, respectively. The PPC2-M group had the fewest OTUs (161) among all the groups.

As reflected in Figure 9B, compared with the NC group, the MC group’s indices were lower. This suggested that the injection of D-galactose led to a drop in the microbial richness in the mice. These indices in the PPC2 and PPC4 (especially PPC4-H) groups were superior to those of the NC group.

As shown in Figure 9C, the intestinal flora of all of the samples was dominated by *Bacteroides* and *Firmicutes* at the phylum level. In contrast to the MC group, the relative abundance of *Bacteroides* in the NC group and the PC group significantly increased, especially in the PPC2-H and PPC4-H groups, 56% and 64%, respectively. The influence of PPC2 and PPC4 on *Bacteroidetes* was dose-dependent.

From Figure 9D, it can be clearly seen that, compared with the blank group at the genus level, the relative abundance of *Muribacelaceae* in the model group decreased, and that of *Lactobacillus* rose. The positive drug groups were significantly more abundant in *Lactobacillus*, but were significantly less rich in *Muribacelaceae*. In the drug groups, PPC2-H and PPC4-H showed the same level of performance. The F + B value of *Firmicutes* and *Bacteroides* in aging mice significantly dropped due to gavage with PPC2 and PPC4, and the values in the model group, the PPC2-H group and the PPC4-H group were 0.93, 0.88 and 0.84, respectively.

## 4. Discussion

In this study, *Hizikia fusiforme* was used as the raw material and subjected to enzyme-assisted water extraction, and two brown extracts, PPC1 and PPC2, were obtained through ethanol precipitation and freeze-drying. The contents of the total phenols, sugars, and sulfate in PPC2 were higher than those in PPC1. In terms of the monosaccharide composition, PPC1 was mainly composed of mannose (81.57%), while PPC2′s main monosaccharide was fucose (51.46%). This was similar to the results of Kong et al. [19]. After oxidization, dialysis and freeze-drying, the pale extracts (PPC3 and PPC4) were obtained. The 30% and 50% reductions in the phenol content in those two products, respectively, were similar to the results reported by Usoltseva et al. [20]. PPC4 contained more fucose, while the other components showed no significant change. The FT-IR results were similar to those of the report by Pawlaczyk-Graja [39]. The absorption peak that appeared at 1270–1260 cm^−1^ indicated the existence of a phenolic sugar complex. The absorption peak at 820 cm^−1^ suggested the existence of a sulfate radical group, similar to the results of Wang et al. [18]. The absorption peak near 2940 cm^−1^ indicated the presence of fucose, similar to the results of Rui et al. [30]. At the level of functional groups, it was further shown that the PPCs had polysaccharide and polyphenol complexes, and it was also found that the polysaccharide contained sulfate and fucose among its monosaccharides [11].

Studies have shown that excessive free radicals are the main cause of aging, and the ability to scavenge free radicals can be assessed by the antioxidant capacity. In this study, we found that PPC had good antioxidant activity in vitro according to four different antioxidant indicators, with PPC4 being the most effective. DPPH is a stable, nitrogen-centered free radical that is widely used to evaluate the antioxidant activity of natural products. Hydroxyl radicals are reactive oxygen species, which can lead to cell damage or death, so it is essential to eliminate excessive hydroxyl radicals. Superoxide anion free radicals are another type of free radical with strong oxidation ability, which can induce lipid oxidation and the decomposition of proteins and polysaccharides. The FRAP assay is a simple and stable test for measuring antioxidant capacity. The different chemical compositions of the extracts were shown to affect the antioxidant activity [40]. In our study, the DPPH radical scavenging activity of PPC1 showed a synergistic effect, which was similar to the study of Obluchinskaya et al. [29]. The FRAP of PPC1 PPC2 had a synergistic effect, which explained the significant decrease in the ferric reducing ability of PPC3 and PPC4 after dephenolization (*p* < 0.01). However, for both hydroxyl radical scavenging and superoxide anion radical scavenging ability, PPC1 and PPC2 showed an antagonistic effect. Obluchinskaya et al. obtained fucoidan from five brown seaweeds and showed synergistic and antagonistic effects in the antioxidant activity, similar to our study [29]. Mercado-Mercado et al. mixed pectin with phenolics, which had an antagonistic effect on antioxidant activity [41]. Airanthi et al., extracted phenolics from Japanese brown seaweed and showed no significant relationship between the total phenol content and antioxidant activity [42]. Antioxidant activity is not always positively correlated with the content of phenolic substances, but is related to the structure and location of the phenolic compounds. The ferric reducing antioxidant power of PPC3 was significantly lower compared with the other three groups of PPCs, likely because of its lower contents of both total sugars and sulfates [26,43]. Studies have shown that the total sugar content is closely related to the antioxidant activity. This was shown by the findings of Wang et al., which indicated that the DPPH and hydroxyl radical scavenging activities of *Hizikia fusiforme* fucoidan with lower levels of total sugars and sulfate were significantly weaker than that of fucoidan, with higher levels of these components [18]. Kong et al. showed that an elevated sugar content in the fractions had a more pronounced effect on the improvement in the intestinal flora of mice [19]. The sulfate content is closely associated with the activity of sulfated polysaccharides. Wu et al. showed that the activity of unsulfated polysaccharide from sea cucumber had better in vitro and in vivo activities compared with the sulfated counterpart [44]. Li et al. used different methods to extract tannin from *Hizikia fusiforme*, and concluded that the products with the highest tannin content had the strongest antioxidant activity [45]. This explains why PPC2 and PPC4, which had a high content of total phenols, sugars and sulfate radicals, had stronger antioxidant activities. The main monosaccharide in these two components was fucose. Meanwhile, previous studies revealed that the fucose content is positively correlated with biological activity [44,46]. Dephenolization of PPC2 produced the more antioxidative PPC4, which may be due to its higher content of fucose and sulfate. As stated in previous studies, polyphenols exist in the fucoidan extraction from *P. polyphylla*, and the presence of polyphenols may affect the extraction and antioxidant activity of high-purity fucoidan.

D-gal (DG) is a reducing sugar existing in various foods. Normal concentrations of DG are metabolized by galactokinase and uridine transferase, while long-term intake of excessive DG will lead to massive amounts of reactive oxygen species (ROS) [47], impairing antioxidant enzyme activities in organs. This will further damage the function of organs and systems in the body.

Various indices in the serum can reflect the degree of aging in the body. The total antioxidant capacity, or T-AOC, can to a certain extent reflect the total capacity to remove reactive oxygen species (ROS) and nitric oxide synthase (NOS) [48]. Glutathione (GSH) and superoxide dismutase (SOD) play important roles in the antioxidant system, and are essential for protecting tissues from oxidative damage [44]. Malondialdehyde (MDA) is one of the products of lipid peroxidation that can cause aging, cell destruction and some diseases [49]. Compared with the normal group, the activities of T-AOC, GSH and SOD in the model group were significantly suppressed, while MDA did not change significantly. After gavage, the activity of antioxidant enzymes was significantly enhanced, and the amount of lipid peroxidation products fell to levels similar to those of the positive drug group, indicating that PPC2 and PPC4 could slow down the aging process in mice. This is consistent with the reports of Yuan et al. [47]. The high-dose group had the best effect.

The liver is an important metabolic organ that can be oxidatively damaged by aging, which produces massive amounts of free radicals. Various indices in the liver can also reflect the degree of aging. CAT catalyzes the decomposition of H_2_O_2_ into H_2_O and O_2_, protecting the structure of cell membranes from peroxides. GSH-PX can also accelerate the decomposition of H_2_O_2_ and reduce the peroxide products to non-toxic hydroxyl compounds [50]. LPO is a product of lipid peroxides, which can reflect the degree of oxidation in mice [51]. Compared with the normal group, the MDA content in the model group increased (*p* < 0.01), the GSH-PX content increased slightly, the LPO clearance rate fell, and the contents of SOD and CAT dropped, similar to the results of Zhao et al. [49]; this indicates that severe oxidative damage had occurred in the liver. Oral gavage of PPC2 and PPC4 boosted the oxidase activity and inhibited lipid peroxidation, and the high-dose group exhibited the best performance. A full analysis of the serum and liver indices showed that PPC4-H had the best inhibitory effect on the oxidative damage caused by D-gal, which may be due to the low content of total phenols, the high content of sulfate, and the high content of fucose in PPC4.

The Nrf2-ARE pathway is the internal mechanism against antioxidant stress [36]. There is a large amount of evidence for the protective role of the Nrf2-ARE pathway in neurodegenerative diseases caused by its ability to mitigate oxidative stress and neuroinflammation. HO-1 is regulated by Nrf2 [52]. Nqo1 is a type of flavinase with antitumor effects, which can inhibit the formation of ROS and inhibit oxidative stress [5]. NOS can control the normal physiological functions in the body. SOD is one of the important antioxidant enzymes in living organisms. Studies have shown that the expression levels of P21 and P16mRNA in senescent cells surge, while the expression levels of GDNF, SCF-1, Nrf2, HO-1 and Nqo1 are significantly downregulated [53]. In this study, D-gal induction knocked down the mRNA and protein expression levels of Nrf2, HO-1, NOS, Nqo1, SOD1 and SOD2 in the brain tissue of mice, which are signs of a successful aging model. The changes in the mRNA expressions of HO-1, Nqo1, NOS, SOD1 and SOD2 in brain tissues after administration of the treatment were consistent with the changes in Nrf2 in a dose-dependent manner, similar to the results of Zhao et al. [5]. Both PPC2 and PPC4 improved the expressions of the Nrf2 pathway proteins, but PPC2′s effects were unstable, with different abilities shown at the medium and high doses. In contrast, PPC4 boasted a more stable performance, and the protein expression levels were enhanced with an increase in the dose. In this regard, PPC4-H took the lead, which was consistent with the mRNA results. It was shown that PPC could affect the expression levels of various antioxidant enzymes in mice by promoting the activation of Nrf2, thereby reducing D-gal-induced senescence.

**Figure 10:**
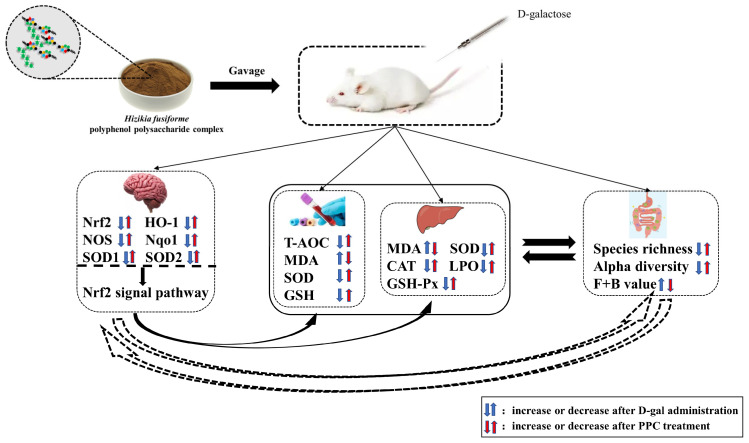
The proposed mechanisms of PPC in ameliorating D-gal-induced aging in mice. Nrf2, nuclear factor erythroid-2-related factor; HO-1, hemeoxygenase-1; NOS, nitric oxide synthase; Nqo1, NAD(P)H quinone oxidoreductase 1; SOD1, superoxide dismutase 1; SOD2, superoxide dismutase 2; T-AOC, total antioxidant capacity; MDA, malondialdehyde; SOD, superoxide dismutase; GSH, glutathione; CAT, catalase; LPO, lipid peroxide; GSH-PX, glutathione peroxidase.

The Human Microbiome Project and human intestinal macrogenics have characterized the human intestinal microbiota, and it was found that the intestinal microbiota consists of archaea, viruses, fungi and other eukaryotes, with *Bacteroides* and *Firmicutes* being the most abundant, while *Proteobacteria* and *Actinomycetes* were found in smaller amounts [54]. Many simulated digestion studies in vitro have shown that plant polysaccharides show insignificant changes in total sugars and molecular weight in gastrointestinal simulations [55,56,57]. In contrast, polysaccharides were significantly degraded during fecal fermentation, showing that intestinal microorganisms can absorb and utilize undegraded polysaccharides. Moreover, polysaccharides can act as functional regulators of the gut microbiota to promote and improve the gut microbiota, and thus protect the host’s health [58]. A growing body of literature has documented the bidirectional communication between the gut microbiota and the brain, called the “gut microbiome–brain axis”. The composition of the intestinal flora changes as the body ages. Key components of the gut microbes, namely *Firmicutes*, *Lactobacillus*, *Bacteroides* and *Aliistipes*, were identified in various proportions in different groups of mice. The aging-related microbiome included “good bacteria”, such as the SCFA-producing bacteria (*Lactobacillus*, *Bacteroides*) and pathogenic bacteria (*Fusobacterium*, *Paracinobacterium*) [59]. In the D-gal intervention group, compared with the normal group, the colony richness and diversity were lower, similar to the results of Spychala et al. [60], who examined the gut microbes in young mice and aging mice, finding that in aging mice, the abundance of thick-walled bacteria and the richness of *Bacteroidetes* increased. This showed that aging will change the composition of the intestinal microbial flora in mice. Analysis of the α diversity of the intestinal microbiota in mice showed that after administration, the α diversity improved significantly, thus improving the diversity of the intestinal microbiota and the evenness of the species [61]. After the administration of PPC, *Bacteroidetes* were enriched in the intestinal tract of mice, indicating that the animals still had strong digestive function despite the aging process, which made the intestinal flora of aging mice younger. The enrichment of *Bacteroidetes* was mainly related to the metabolism of polysaccharides, while the *Firmicutes* include a small number of polysaccharide-degrading enzymes. As absorption is related to the intestinal microbial metabolism, the improvement in polysaccharide metabolism increased the digestive ability of the small intestine, but the aging process would lead to slowing of polysaccharide metabolism [62]. There were no significant differences between PPC2 and PPC4 in the abundance of intestinal microflora in aging mice. To a certain extent, they could rejuvenate the intestinal flora of aging mice. In summary, in Figure 10 we map the mechanisms to interpret the pathways through which PPC ameliorates D-galactose-induced aging in mice.

## 5. Conclusions

Two polyphenol–polysaccharide complexes, PCC1 and PCC2, were prepared from *Hizikia fusiforme* via enzyme-assisted extraction. They showed significant differences in their compositions of total sugars, total phenols, sulfate and monosaccharides. PCC3 and PPC4 were effectively prepared via treatment with H_2_O_2_. Phenols could be removed from PPC1 and PPC2 without marked changes in the compositions of total sugars, sulfate and monosaccharides. Dephenolized PPC4 boasted better antioxidant activity in vitro, and could inhibit the intensified oxidation and the weakening of the activity of antioxidant enzymes caused by D-gal, activate the Nrf2-ARE signal pathway, and diversify the intestinal flora, and it showed obvious anti-aging activity. The structure–activity relationships of natural and modified polyphenol–polysaccharide complexes in *Hizikia fusiforme* are worth focusing on in future research. Above all, PPC4 represents a new prospect for the development of functional food and medicine.

## Figures and Tables

**Table 1:** Primer sequences used for real-time RT-PCR.

Gene	Forward	Reverse
HO-1	TAGAGCGCAACAAGCAGAAC	ATGATTTCCTGCCAGTGAGG
NOS	CAGCTGGGCTGTACAAACCTT	CATTGGAAGTGAAGCGTTTCG
Nqo1	TTACAGCATTGGCCACACTC	GGCTGCTTGGAGCAAAATAG
Nrf2	TTCTTTCAGCAGCATCCTTCTCCAC	ACAGCCTTCAATAGTCCCGTCCAG
SOD1	AGATGACTTGGGCAAAGGTG	AATCCCAATCACTCCACAGG
SOD2	CTGGCTTGGCTTCAATAAGG	TAAGGCCTGTTGTTCCTTGC

Note: Nrf2, nuclear factor erythroid-2-related factor 2; HO-1, hemeoxygenase-1; NOS, nitric oxide synthase; Nqo1, NAD(P)H quinone oxidoreductase 1; SOD1, superoxide dismutase 1; SOD2, superoxide dismutase 2. All primers were purchased from Acres Biological Engineering Co., Ltd., (Hunan, China).

**Table 2:** Composition of each group of extracts of Hizikia fusiforme (dry weight %).

Component	Monosaccharide Composition (%)								Total Phenol (%)	Total Sugar (%)	Sulfate (%)	Yield (%)
	Fuc	Gal	Glu	Gal-UA	Glu-UA	Man	Rha	Xyl				
PPC1	11.39	2.11	2.81	3.11	0.73	76.16	2.67	1.09	1.10 ± 0.02	31.12 ± 0.20	4.79 ± 0.14	24.22 ± 3.78
PPC2	64.52	11.05	17.1	1.83	0.76	2.56	0.77	1.49	3.11 ± 0.20	41.33 ± 0.18	13.18 ± 0.27	11.87 ± 2.33
PPC3	10.41	2.09	2.99	3.04	1.09	76.56	3.84	1.75	0.77 ± 0.01	27.67 ± 0.46	3.86 ± 0.10	14.53 ± 1.24
PPC4	68.03	11.89	9.35	4.63	0.74	2.99	1.17	1.82	1.62 ± 0.08	37.85 ± 0.31	13.59 ± 0.33	7.12 ± 0.45

Note: PPC1 and PPC2 were polyphenol–polysaccharide complexes obtained from ethanol precipitation using 30% and 70% ethanol, while PPC3 and PPC4 were the oxidated and dialyzed products of PPC1 and PPC2, respectively. Fuc, fucose; Gal, galactose; Man, mannose; Glu, glucose; Rha, rhamnose; Xyl, xylose; Glu-UA, glucuronic acid; GLA-UA, galactose acid.

## Data Availability

The data used to support the findings of this study can be made available by the corresponding author upon request.
